# Alternative Splicing: Recent Insights into Mechanisms and Functional Roles

**DOI:** 10.3390/cells9102327

**Published:** 2020-10-20

**Authors:** Claudia Ghigna, Maria Paola Paronetto

**Affiliations:** 1 Istituto di Genetica Molecolare Luigi Luca Cavalli Sforza—Consiglio Nazionale delle Ricerche, via Abbiategrasso 207, 27100 Pavia, Italy; 2 Department of Movement, Human and Health Sciences, University of Rome “Foro Italico”, 00135 Rome, Italy

**Keywords:** alternative splicing, RNA binding proteins, human diseases

Alternative splicing generates multiple protein isoforms from one primary transcript and represents one of the major drivers of proteomic diversity in human cells [1]. Alternative splicing variants regulate cell-, tissue- or developmental-specific programs, whereas their aberrant expression is involved in many pathologies, including cancer [2,3]. In this regard, alternatively spliced isoforms expressed exclusively in tumor cells are particularly relevant for the diagnosis, prognosis and targeted therapy of multiple cancer types [4]. 

Although significant progress has been made in recent years, important questions still remain to be addressed. In particular, we need to determine (i) the role(s) of the majority of the splicing isoforms during physiological (or pathological) processes, (ii) the factors involved in their production, and (iii) how splicing is integrated with other gene expression regulatory programs.

This special issue collects recent insights addressing the above interrogatives. More specifically, Palombo and colleagues illustrate the function of two splicing factors (hnRNPM and SRSF3) in regulating DHX9 poison exon, with potential implications in Ewing sarcoma proliferation and sensitivity to chemotherapy [5], whereas Gajan and collaborators highlight splicing errors in *RAD6B* gene and their association with melanoma pathogenesis [6]. La Cognata and coworkers describe the involvement of splicing factors and spliceosome components in the pathogenesis of Amyotrophic Lateral Sclerosis (ALS) [7], while Mfossa et al. identify circular RNAs induced in response to radiation in a p53-dependent fashion [8], which may represent biomarkers of brain ageing. Moon and collaborators deal with the splicing regulation and activation of a cryptic 3’ splice site in *SMN2* gene [9], involved in another human disorder, such as the Spinal Muscular Atrophy (SMA). NOVA2 regulation of splicing isoforms for two transcription factors (Ppar-γ andTfdp2) and the subsequent control of mRNA steady-state levels in endothelial cells is presented by Belloni et al. [10]. In their article, Yu and colleagues use proteomics data to validate and modify the gene annotation information of *moso bamboo* (an important forest species) by supporting the translation of a fraction of transcript isoforms targeted by nonsense-mediated mRNA decay (NMD) pathway [11]. Taking advantage of high throughput RNA sequencing approaches, Neves-da-Rocha and colleagues identify intron retention events in the transcripts encoding Hsp70 family members and discuss the role played by the regulation of HSP-mediated networks in cell adaptation in *Trichophyton rubrum* (a dermatophytic fungus) suggesting strategies employed by dermatophytes in response to antifungal therapy [12].

This special issue also includes four reviews. In particular, Biamonti and colleagues discuss the key role played by alternative splicing in cancer cell plasticity and tumor heterogeneity [13]. The possibility of using alternative splicing as a prognostic factor and potential therapeutic target in cancer is addressed by Nikas et al., which discuss the prognostic role of SRPK1 (an enzyme that phosphorylates splicing factors rich in serine/arginine domain) and preclinical studies supporting SRPK1 as cancer treatment target are presented [14]. Bielli et al. focus on the role of alternative splicing errors in brain tumors and present recent efforts aimed at developing novel splicing-targeted cancer therapies [15]. Finally, Rowlands and colleagues present a critical comparative analysis of the bioinformatic tools designed to predict genomic variants impacting on the splicing process [16]. 

We hope that the articles and reviews included in this special issue will illuminate new progresses in the field of alternative splicing and its function(s) in physiological and pathological conditions.
